# Vector-free transmission and persistence of Japanese encephalitis virus in pigs

**DOI:** 10.1038/ncomms10832

**Published:** 2016-02-23

**Authors:** Meret E. Ricklin, Obdulio García-Nicolás, Daniel Brechbühl, Sylvie Python, Beatrice Zumkehr, Antoine Nougairede, Remi N. Charrel, Horst Posthaus, Anna Oevermann, Artur Summerfield

**Affiliations:** 1 Institute of Virology and Immunology, Sensemattstrasse 293, Mittelhäusern, Bern 3147, Switzerland; 2 Aix Marseille Université, U190-IRD French Institute of Research for Development, U1207-INSERM Institut National de la Santé et de la Recherche Médicale, EHESP French School of Public Health, EPV UMR_D 190 “Emergence des Pathologies Virales”, & IHU Méditerranée Infection, APHM Public Hospitals of Marseille, 27 Boulevard Jean-Moulin, Marseille 13385, France; 3 Institute for Animal Pathology, Vetsuisse faculty, University of Bern, Länggassstrasse 122, Bern 3001, Switzerland; 4 Division of Experimental Clinical Research, Vetsuisse Faculty, University of Bern, Bremgartenstrasse 109a, Bern 3001, Switzerland; 5 Department of Infectious Diseases and Immunopathology, Vetsuisse Faculty, University of Bern, Länggassstrasse 122, Bern 3001, Switzerland

## Abstract

Japanese encephalitis virus (JEV), a main cause of severe viral encephalitis in humans, has a complex ecology, composed of a cycle involving primarily waterbirds and mosquitoes, as well as a cycle involving pigs as amplifying hosts. To date, JEV transmission has been exclusively described as being mosquito-mediated. Here we demonstrate that JEV can be transmitted between pigs in the absence of arthropod vectors. Pigs shed virus in oronasal secretions and are highly susceptible to oronasal infection. Clinical symptoms, virus tropism and central nervous system histological lesions are similar in pigs infected through needle, contact or oronasal inoculation. In all cases, a particularly important site of replication are the tonsils, in which JEV is found to persist for at least 25 days despite the presence of high levels of neutralizing antibodies. Our findings could have a major impact on the ecology of JEV in temperate regions with short mosquito seasons.

## Abstract (web summary)

Japanese encephalitis virus (JEV) is primarily transmitted between mosquitoes and birds but can also infect pigs. Here the authors demonstrate that JEV, which was thought to be spread exclusively by mosquitoes, can be transmitted between pigs through a direct contact.

## Graphical abstract

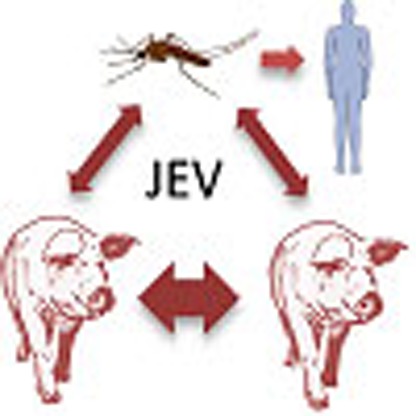

Japanese encephalitis virus (JEV) causes an important zoonotic vector-borne disease first isolated from a human in Japan in 1935 (ref. 1). It is currently present in East and Southeast Asia and Australia^2^,^3^. The annual incidence of human cases is reported to be in the range of 50,000–175,000 (refs 2, 3, 4). During an epidemic, roughly 0.1–4% of infected individuals develop clinically apparent encephalitis. The mortality associated with JEV encephalitis is as high as 25–30%, and ∼50% of surviving patients suffer neuropsychiatric sequelae^2^,^3^. JEV is considered to be the most frequent viral encephalitis associated with fatal or severe outcomes^5^.

JEV is vector-borne with *Culex* mosquitoes as its main vectors, and with waterbirds such as egrets and herons as reservoirs. However, it has been reported that pigs serve as amplifying hosts in human epidemics^2^,^3^,^6^,^7^,^8^,^9^,^10^. As early as the 1950s, studies found that pigs are readily infected with JEV and develop viraemia for several days^8^,^11^. While clinical symptoms in pigs are mild, humans and horses can develop severe disease with encephalitis. Factors favouring pigs as being the main amplifying host for JEV are a high birth rate and a rapid population turnover, resulting in constant generation of an immunologically naive population. Furthermore, an important JEV vector *Culex tritaeniorhynchus* preferentially feeds on pigs^2^,^3^,^9^,^12^,^13^. Fortunately, viraemia in humans and horses is probably insufficient to infect mosquitoes, and they are considered to be dead-end hosts^2^.

In the past, the temperate northern Japanese Island Hokkaido was affected by JEV epidemics, and the virus was shown to hibernate and re-emerge in the same local region^14^. Some ecological and epidemiological aspects of these outbreaks remained enigmatic. First, during several Japanese encephalitis outbreaks, no virus was isolated from locally collected mosquitoes^15^,^16^. Second, two geographically isolated distinct outbreaks were identified on pig farms in Hokkaido over at least 3 years, demonstrating that the virus can hibernate locally^14^. However, the underlying mechanisms were not clarified. Also, during epidemiological investigations in Taiwan, no viraemic mosquitoes were found in the period before JEV outbreaks in pigs^17^.

We questioned if vector-free transmission might be possible and, if so, could help explain some of the observations made in temperate regions. Therefore, in the frame of a pathogenesis study with pigs, we placed sentinels with intravenously (i.v.) infected pigs and found vector-free transmission of JEV in pigs. This finding was confirmed and further supported by demonstrating efficient oronasal infection with low doses of virus. Moreover, tonsils appear to play a prominent role as a source of virus replication and persistence.

## Results

### JEV can transmit between pigs in the absence of vectors

**Figure 1:**
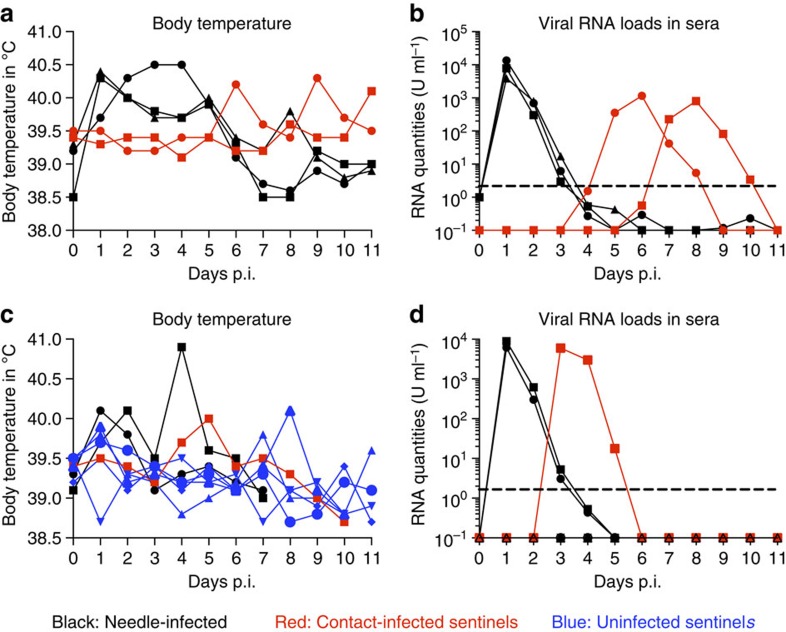
Body temperature and viraemia of pigs infected by needle or contact. (**a**,**b**) Three needle-infected pigs (10^7^ TCID_50_ per pig, black lines) were housed with two naive sentinel animals (red lines). (**c**,**d**) Two animals were needle-infected (black) and housed with six sentinel pigs (red and blue). Viraemia is shown as viral RNA loads determined by real-time RT–PCR, and expressed as U ml^−1^ (1 U corresponding to the RNA quantity found in 1 TCID_50_ of a virus stock).

We observed JEV transmission from needle-infected pigs to uninfected naive pigs when three infected pigs were housed with two uninfected animals. Before infection, all piglets were healthy and alert, with normal body temperatures of 38.7–39.4 °C. Body temperature in the needle-infected animals increased after 24 h, with readings up to 40.6 °C; fever lasted for 4–5 days before dropping to pre-infection levels (Fig. 1a). The two contact animals developed fever 6 and 9 days after needle infection of the other three animals. In all but one animal, fever curves were double-peaked. Appetite was reduced in all animals. They produced less manure and were reluctant to move for 3–6 days. When body temperature normalized, clinical symptoms declined and finally disappeared.

Viraemia in needle-infected animals lasted 3 days and reached maximum values in the range of 10^4^ RNA U ml^−1^ (Fig. 1b); viral titres were 3.2 × 10^4^ (2 animals)–3.2 × 10^5^ (1 animal) tissue culture infectious dose 50 (TCID_50_) per ml. Viraemia was found in both contact pigs for 2 and 4 days, with maximum values around one to two orders of magnitude lower than the maximum in the needle-infected animals (Fig. 1b). Both sera were positive for virus isolation by cell culture, but their infectious titres were close to the detection limit of the assay (50 TCID_50_ per ml). To verify this unexpected and to our knowledge previously not described observation of viral transmission in a vector-free environment, we conducted a second experiment. Two animals were needle-infected and six healthy animals were kept in the same stable to act as sentinels. Clinical outcomes in the needle-infected animals were as described above, with one pig's body temperature passing 41 °C. Both became viraemic both in terms of viral RNA (Fig. 1c,d) and live virus detection (1.5 and 3.2 × 10^4^ TCID_50_ per ml). Body temperature increased over 40 °C in two of the sentinels (Fig. 1c). However, real-time reverse transcription–quantitative PCR (RT–qPCR) revealed viraemia in only one. Viraemia lasted for 3 days, and values were close to 10^4^ RNA U ml^−1^, similar to the needle-infected pigs (Fig. 1d). The other five sentinel animals did not develop detectable viraemia or seroconversion, and RT–qPCR-positive organs were not detected during the 11-day observational period.

### JEV organ tropism is independent of mode of infection

**Figure 2:**
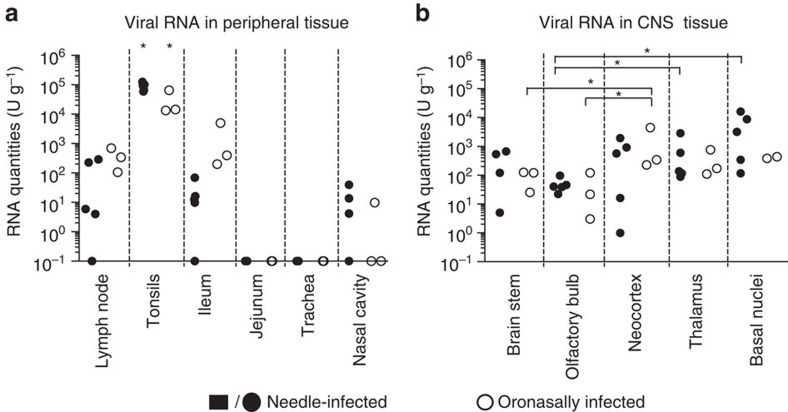
Viral RNA loads in peripheral and CNS tissues. Viral RNA loads in peripheral (**a**) and CNS (**b**) tissues were determined at necropsy by real-time RT–PCR and expressed as U g^−1^ (1 U corresponding to the RNA quantity found in 1 TCID_50_ of a virus stock). Solid symbols represent needle-infected pigs (*n*=5) killed at 7 (circles) and 11 (squares) days p.i. Open symbols represent pigs infected by contact (*n*=3). Two animals, corresponding to those shown in Fig. 1b, were killed at day 11, which was 4 and 7 days after the peak of viraemia, respectively. One animal, corresponding to Fig. 1d, was killed at day 10, which was 7 days after peak viraemia. Asterisks (*) indicate significant differences calculated with a nonparametric two-tailed Mann–Whitney *U*-test (*P*<0.05).

Necropsy of needle-infected animals was performed at day 11 (first experiment) or 7 (second experiment), and necropsy for sentinels was performed at day 10 or 11, which was 6–8 days after estimated transmission (post transmission). Figure 2 shows relative RNA quantities for both needle-infected and sentinel animals. Relative RNA quantities were comparable between all animals, independent of mode of infection. The lymph nodes, the ileum with its continuous Peyer's patches, and parts of the nasal cavity were positive for viral RNA. Interestingly, relative RNA levels of up to 10^5^ U g^−1^ were found in the tonsils. These values were 1–2 orders of magnitude higher than in the other organs (Fig. 2a). In the brain tissues, values were also comparable between the needle- and contact-infected pigs. All examined regions remained positive until the end of the study period, with highest levels of up to 10^3^–10^4^ RNA U g^−1^ in the (frontal) neocortex, thalamus and basal nuclei. In the brain stem and olfactory bulb, we found roughly 10 times less viral RNA compared with the other regions (Fig. 2b). By titrating lysed material from the tonsils, we confirmed the presence of live virus in all tonsils of infected pigs (Table 1).

### Oronasal virus shedding by JEV-infected pigs

**Figure 3:**
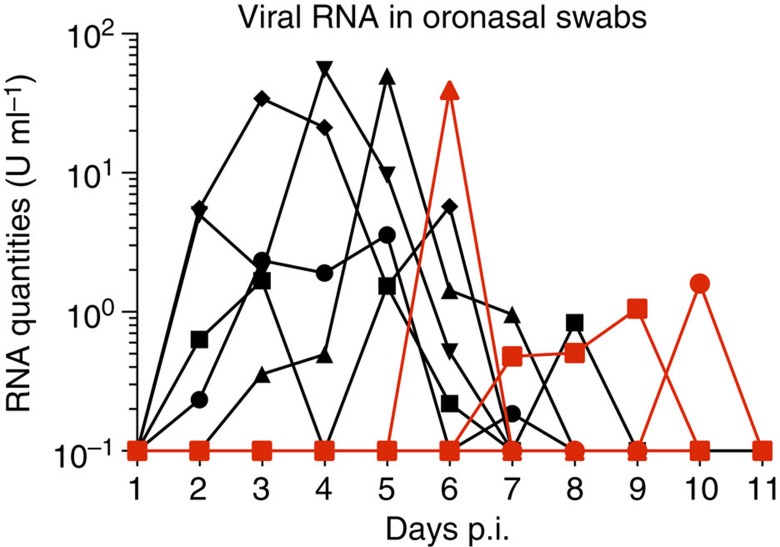
Viral RNA load in oronasal swabs. Oronasal swabs were collected daily from the experiments shown in Fig. 1 (experiment one and two as described in Methods); viral loads were quantified by real-time RT–qPCR (1 U corresponding to the RNA quantity found in 1 TCID_50_ of a virus stock). Black: needle-infected animals (*n*=5); red: contact-infected pigs (*n*=3).

RT–qPCR indicated that needle-infected animals started to shed virus oronasally as early as two days post infection (p.i.) for a period of ∼4 days. Animals infected by contact first shed virus 5 days after first contact with the needle-infected animals. In two of them, viral RNA in oronasal swabs was detected for 1 day only. The third animal shed virus for 3 days (Fig. 3). Swabs from the eyes, rectum and vagina/preputium, and the urine were negative, with the exception of one animal in which a foreskin swab was RT–qPCR positive at 5 days p.i. (0.6 RNA U ml^−1^). We used cell culture to confirm that the oronasal swabs contained live virus. Most swabs collected at 4–5 days p.i. were positive (Table 2, second column). Similarly, pigs infected by contact shed live virus 6–10 days after contact, depending on the animal (Table 2, third column).

### Pigs are highly susceptible to oronasal JEV infection

**Figure 4:**
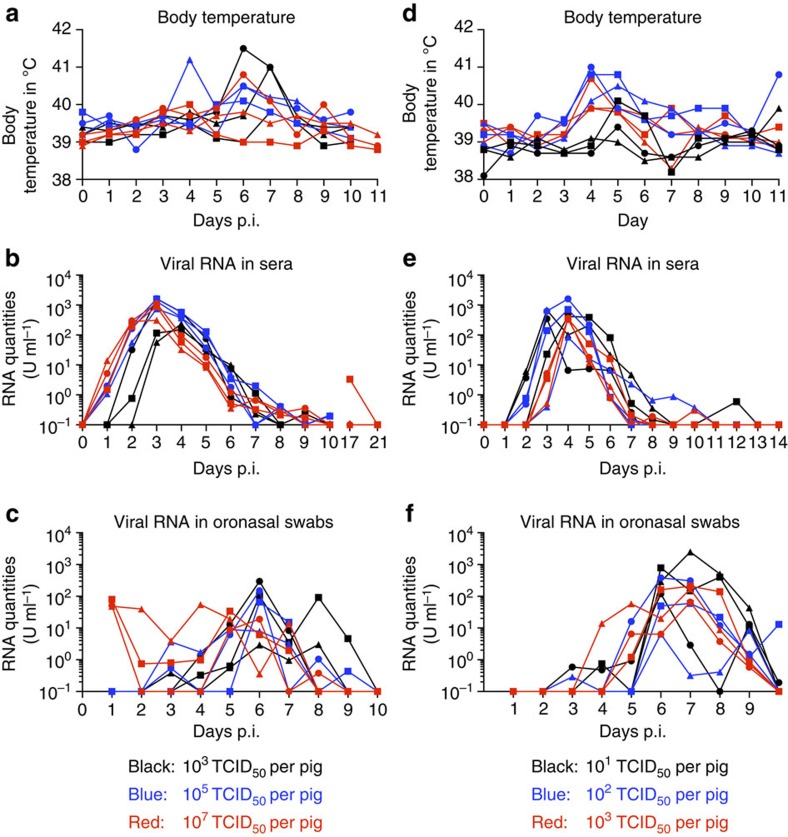
Body temperature and viral RNA loads in serum and swabs of oronasally infected pigs. In **a**–**c**, groups of three animals were infected oronasally with JEV at 10^3^ (black), 10^5^ (blue) or 10^7^ (red) TCID_50_ per pig. (**d**–**f**) A second experiment with groups of three animals infected oronasally at 10^1^ (black), 10^2^ (blue) or 10^3^ (red) TCID_50_ per pig. Viral RNA loads in serum (**d**,**e**) and oronasal swabs (**c**,**f**) were determined as for the other figures, and are expressed as U ml^−1^.

Considering that three out of eight in-contact animals became ill and shed virus oronasally for 1–4 days, we tested the oronasal route as a means of infection by JEV. As described in Methods, nine animals were infected oronasally using three different doses of virus. In all nine pigs, body temperatures raised after 4–9 days, reaching 41.5 °C in some animals. Interestingly, two animals infected with the lowest dose (10^3^ TCID_50_) developed the highest body temperatures. By day 10 p.i., body temperatures of all animals returned to normal levels (Fig. 4a). Viraemia in all three groups was comparable, although two animals infected with the lowest dose developed viraemia 1–2 days later than the other pigs (Fig. 4b). In all pigs, viraemia lasted for 4 days. One animal, infected with the highest dose, suffered from rebound viraemia on day 16 p.i., with viral RNA detected in the serum. Only one blood sample per week was taken, and we cannot determine the duration of this second viraemia.

Animals infected via the oronasal route were also positive for JEV RNA in oronasal swabs (Fig. 4c). Swabs were positive in pigs infected with the highest dose at day 1 p.i., possibly representing input virus. Thereafter, most pigs shed virus between day 4 and 7 p.i., with relative RNA levels reaching 100 U ml^−1^. Nevertheless, some animals had RNA-positive swabs up to 9 days p.i., which was 2 days beyond the end of the viraemic phase (Fig. 4c). Between 3 and 6 days p.i., the majority of swabs were also positive for virus isolation (Table 2).

Considering the relatively low levels of virus in oronasal swabs, we decided to perform a follow-up oronasal experimental infection with doses of 10, 100 and 1,000 TCID_50_ per pig (Fig. 4d,e). Strikingly, all animals again became infected, with incubation times of 2–3 days and viraemia lasting 5–6 days. At 4 and 5 days p.i., body temperature in all pigs was above 39.5 °C (Fig. 4d). Again, as early as 3 days p.i., some pigs had viral RNA-positive oronasal swabs; by day 7 all swabs were positive (Fig. 4f). In most pigs, oronasal virus excretion lasted 5–6 days.

**Figure 5:**
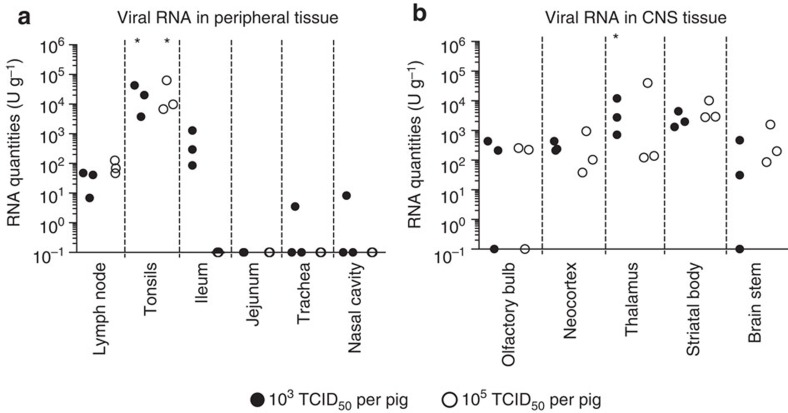
Virus tropism in oronasally infected pigs. Viral load in peripheral organs (**a**) and CNS (**b**) of oronasally infected pigs was assessed at 10 days p.i. by real-time RT–PCR, and shown in relative RNA quantities (U ml^−1^). Animals were infected with either 10^3^ (filled circles) or 10^5^ (open circles) TCID_50_. Asterisks (*) indicate significant differences calculated with a nonparametric two-tailed Mann–Whitney *U*-test (*P*<0.05).

Viral tropism for non-central nervous system (CNS) tissue was similar in needle and oronasally infected pigs. At necropsy 10 days p.i., lymph nodes, ileum and tonsils from the lowest-dose-infected pigs were positive for viral RNA (Fig. 5a). In the lymph nodes and ileum, only 100–1,000 RNA U g^−1^ were detected, while almost 100,000 RNA U g^−1^ were found in the tonsils. No difference was observed between the 10^3^ and 10^5^ dose in the lymph nodes and tonsils. The trachea and nasal cavity were negative for viral RNA except in one pig (Fig. 5a). Urine samples were collected on the day of slaughter; we found one positive sample (0.5 RNA U ml^−1^). RNA levels in the brain were comparable in tissues isolated from pigs infected with the low and middle doses. Thalamus and basal nuclei reached the highest levels of around 1,000–10,000 RNA U g^−1^ (Fig. 5b).

### Histopathological lesions

**Figure 6:**
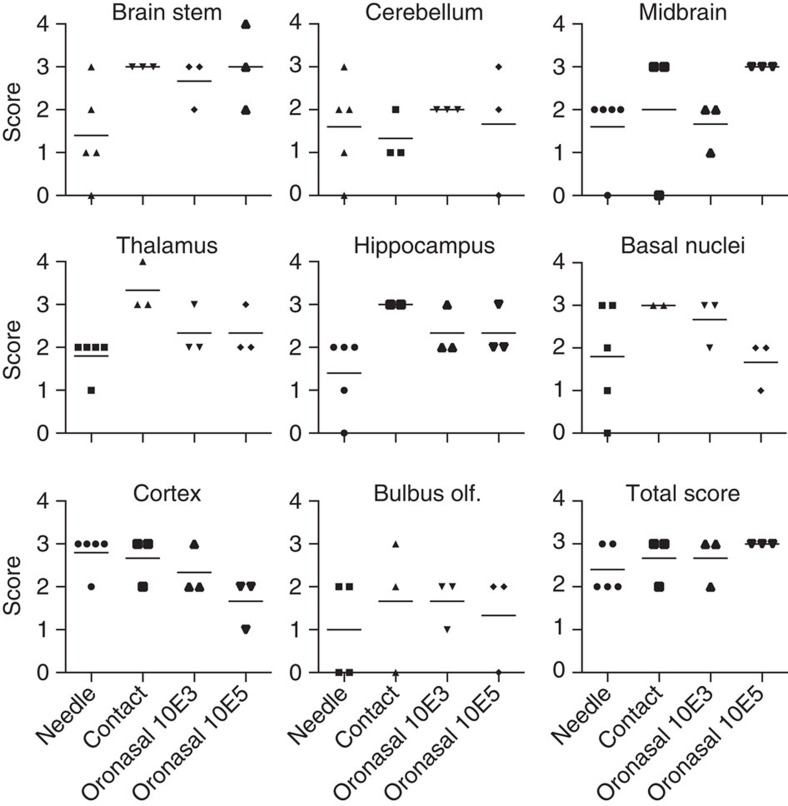
CNS lesions in pigs are not dependent on the route of infection. Lesions in the central nervous system were semiquantitatively scored from 0 to 4 (0: no lesions; 1: minimal lesions; 2: mild lesions; 3: moderate lesions; 4: severe lesions) as described in the Methods section. The needle-infected group (needle) was analysed 6 and 10 days after the onset of viraemia (taken as a measure due to the unknown infection time and different incubation periods of the other groups). Tissue from pigs infected by contact (contact) was 4, 6 and 7 days after the onset of viraemia. Tissue from oronasally infected animals was 7, 8 and 9 days after the onset of viraemia. CNS tissue from the animals infected with 10^3^ TCID_50_ (oronasal 10E3) and 10^5^ TCID_50_ (oronasal 10E5) is shown. olf., olfactory.

Regardless of mode of infection, JEV induced histopathological CNS lesions typical of a viral meningoencephalomyelitis. Lesions were characterized by multifocal lymphohistiocytic perivascular cuffs affecting mainly the grey matter, and to a lesser degree, the white matter. They were associated with glial nodules and evidence of neuronal degeneration and necrosis. Frequently, few neutrophils were present in the areas of neuronal necrosis. In addition, multifocal mild lymphohistiocytic meningitis was present. Scoring the lesions in the brain stem, cerebellum, midbrain, thalamus, hippocampus, basal nuclei, neocortex and the bulbus olfactorius indicated that the mode of infection did not fundamentally influence virus-induced pathology and distribution of CNS lesions (Fig. 6). Note that the time after the virus had reached the CNS was unequal between the groups as the animals were not slaughtered the same day, the incubation period differed (Figs 1 and 4) or the time of infection was unknown. Nevertheless, the overall score calculated as an average score of the CNS tissue analysed was similar for all modes of infections. Lymphatic tissues including tonsils showed slight follicular hyperplasia, which is indicative of activation but otherwise no pathological alterations.

### JEV can persist in the tonsils for at least 25 days

**Figure 7:**
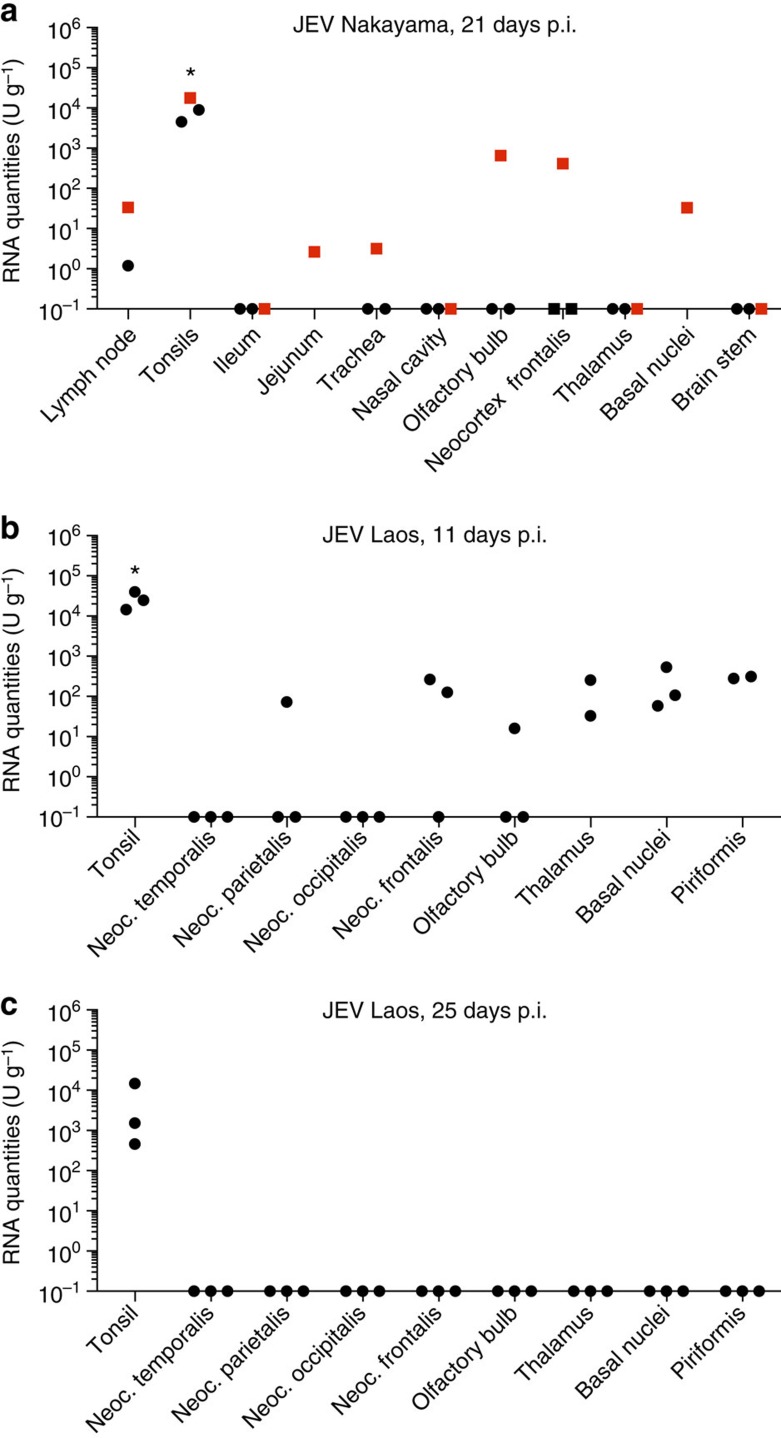
Persistence of JEV in the tonsils. (**a**) Various tissues were collected from oronasally infected pigs (10^7^ TCID_50_, Nakayama strain) and analysed for viral RNA load by real-time RT–PCR. The animal depicted by red squares is identical to the animal depicted in Fig. 4b, which became viraemic at day 17 p.i. but cleared virus from the blood by day 21 p.i. (**b**,**c**). Six pigs were needle-infected with the Laos strain of JEV, and tonsils were analysed for viral RNA load at 11 and 25 days p.i. Viral RNA loads were determined as for the other figures and expressed as U ml^−1^. Asterisks (*) indicate significant differences calculated with a nonparametric two-tailed Mann–Whitney *U*-test (*P*<0.05). Neoc., neocortex.

The levels of viral RNA were always highest in the tonsils in all animals, independent of the route and dose of infection (Figs 2 and 5). Given that the longest observational time in our initial experiment was 11 days p.i., we decided to keep a new group of animals longer to examine the potential persistence of JEV. Strikingly, on day 21 after oronasal infection with the Nakayama strain, the peripheral organs and CNS for two animals were negative for viral RNA, but 10^3^–10^4^ U g^−1^ remained in the tonsils. These values were comparable to those at 7 and 11 days p.i. The lymph nodes, jejunum, trachea, olfactory bulb, neocortex and basal nuclei were positive in one animal (Fig. 7a, red squares). This animal had a second viraemia at 17 days p.i. (Fig. 4b). RT–PCR was negative in urine samples collected at 21 days p.i.

To determine if persistence of virus in the tonsils is unique to the Nakayama strain or if it can result from infection with other genotypes, we analysed six pigs infected with Laos strain, a genotype I JEV. At necropsy on day 11, the highest RNA values were in the tonsils, whereas the CNS samples were negative or roughly two orders of magnitude lower (Fig. 7b). Strikingly, at 25 p.i., all organs were negative for viral RNA except the tonsils, confirming JEV's ability to persist in this organ for over 3 weeks (Fig. 7c). All tonsils of infected pigs were also positive for virus isolation.

### Immune response

**Figure 8:**
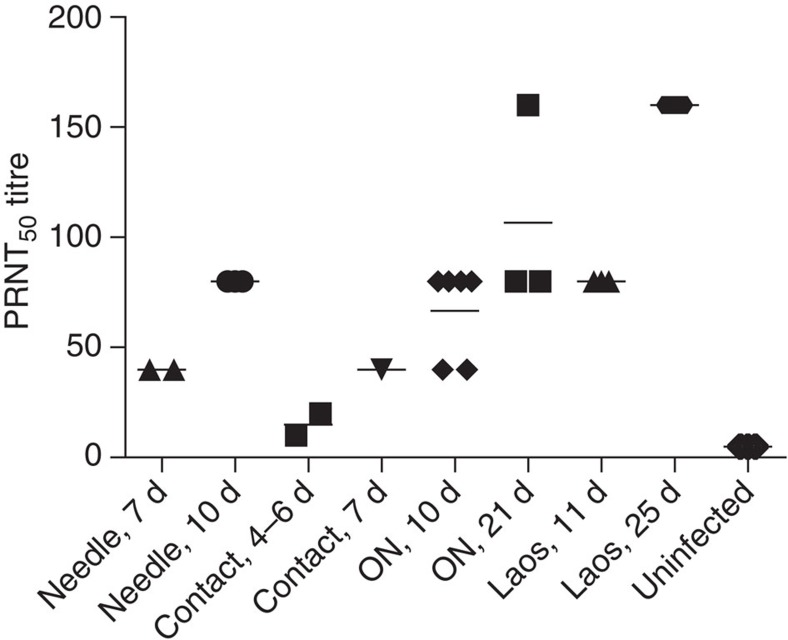
Virus neutralization titres. Sera obtained from experimentally infected groups were obtained at the indicated days (d) p.i. and tested in a PRNT test. Needle: animals infected with the Nakayama strain (Fig. 1); contact: animals infected by contact (see Fig. 1, time point p.i. is estimated); ON: pigs infected via the oronasal route (Fig. 4); Laos: pigs infected i.d. with the Laos strain of JEV (10^6^ TCID_50_).

All infected animals mounted a rapid immune response in terms of JEV-neutralizing antibodies (Fig. 8). At 7–10 days p.i. (6–9 days after first viraemia), all animals had titres of 40–80 TCID_50_ per ml. Similar titres were found in pigs infected by contact. This finding was confirmed in the oronasally infected pigs. The levels of neutralizing antibodies increased with time after infection, but they did not differ by mode of infection or JEV genotype. These results demonstrate that JEV persists in the tonsils despite the presence of an efficient humoral immune response.

## Discussion

This study describes two findings concerning JEV infection in pigs, both of which may have a significant impact on our understanding of JEV's ecology, epidemiology and on approaches to controlling it. First, vector-free transmission between pigs can occur via direct contact, with animals being highly susceptible to oronasal infection. Second, the tonsils are a primary replication site of JEV, regardless of mode of infection, and JEV can persist in them for at least 25 days despite the presence of neutralizing antibodies.

Textbooks and published scientific articles describe Japanese encephalitis as being exclusively mosquito-borne, with *Culex* species as the main vectors (reviewed in refs 2, 3). In our first experimental infection, both sentinel animals became ill, but only one out of six was infected in the second. This difference could be due to the fact that in the second experiment only two animals were needle-infected, which could have reduced the chances of contact. In fact, our facility's efficient ventilation system and low stocking density (>3 m^2^ per animal) support transmission by contact-dependent route rather than by aerosols. Although our study had too few animals to estimate the reproduction value of transmission, it indicates that this process is not as efficacious as with viruses that have adapted to enter through the mucosal surfaces of the airways, such as influenza virus. This possibility is understandable, given that mosquitoes are clearly the main transmission mode of JEV. Nevertheless, it is possible that under field conditions with a dense pig population and other pathogens, the rate of vector-free transmission could be higher compared with experimental conditions with clean stables, controlled temperature and humidity, high ventilation and no crowding. Furthermore, with our experimental conditions and specific-pathogen-free (SPF) status, our pigs are indeed relatively resistant to pathogenic virus infections.

Certainly, our data demonstrate that pigs are highly susceptible to oronasal infection with JEV, as a dose of only 10 TCID_50_ per animal was sufficient to infect all animals in this group. Interestingly, in a *Rhesus macaque* model used to test JEV vaccines, nasal infection was also used^18^,^19^,^20^. In that experiment, doses causing disease were much higher with 6.6 × 10^6^–2 × 10^10^ infectious units per animal required for infection. One study found that mice are also susceptible to oral infection with a dose of 1–2 × 10^7^ infectious units per animal, although in that study, no virological data was published^21^.

Importantly, although the levels of viral RNA and live virus isolated from oronasal swabs was low, mucosal virus shedding lasted up to 6 days in some animals, indicating that this could be the relevant source of virus for transmission. Our viraemia data indicate that the incubation period for pigs infected by contact was 3–5 days with respect to the development of viraemia. The incubation period was only 1 day when high oronasal virus doses were used (10^5^ and 10^7^ infectious units), but 2–3 days with low doses (10–1,000 units). This short incubation time could indicate that the total amount of virus transmitted by contact between pigs and resulting in an infection can be below 10 infectious units.

Different modes of infection did not result in fundamental differences in viraemia, virus excretion through the upper respiratory tract, virus tropism in the lymphoid and CNS tissues, and antibody response. If anything, viraemia lasted longer after oronasal/contact transmission. The virus doses employed for oronasal inoculation also did not appear to have a major impact on these parameters. The viraemia we observed is similar to that found by others, reaching levels of ∼10^4^ infectious units per ml (refs 7, 22, 23, 24). This level appears to be sufficient to transmit the virus to mosquitoes. For example, Takahashi *et al*.^25^ found that 50% of mosquitoes ingesting 50 LD_50_ became infected. Thus, assuming a blood meal of 2 μl, it can be expected that a low level of viraemia would transmit virus to significant numbers of mosquitoes. Other work has demonstrated that viraemic levels comparable to those found here were sufficient to transmit virus to up to 33% of mosquitoes^22^.

Considering the high viral load in the tonsils, the lymphoid tissue of the oropharynx could be a possible source of virus leading to oronasal infection. Also, the peak of viral RNA in oronasal swabs was found around 6 days p.i., which was 2–3 days after the peak of viraemia. In addition, virus-positive swabs were still found after the viraemic phase.

Several reports have demonstrated oral or nasal infection of West Nile virus (WNV), a closely related flavivirus, in a wide range of different species, including mice, wild birds, hamsters and alligators^26^,^27^,^28^,^29^. In humans, there is evidence for transmission via breastfeeding; this evidence is supported by data in hamsters^27^,^30^. Furthermore, laboratory infections may have occurred through aerosol transmission^31^,^32^. Although data supporting oronasal JEV infection species other than pigs is rare, our data should be taken as a warning that infection via the oronasal route might be possible, and direct pig–human and bird–bird transmissions cannot be excluded. Clearly, these possibilities require future investigation.

Our second important observation was JEV's tropism for the tonsils, where viral loads were 2–3 orders of magnitude higher than in other organs. Furthermore, JEV can persist in the tonsils for at least 3 weeks. To our knowledge, JEV infection of the tonsils in other species has not been described, although in one study, the tonsils were used as a source of virus isolation in pigs^33^. We found that high viral load in the tonsils persisted well beyond the viraemic phase of infection, despite the presence of neutralizing antibodies. Even by week 3 p.i., high RNA and live virus levels were detected in tonsil homogenates. All other organs tested were negative by then.

As this finding indicates a possible persistence for more than 1 month, future studies are required to determine the occurrence and duration of JEV persistence in porcine tonsils under field conditions. Persistence may be associated with reactivation and oronasal transmission events to naive pigs, thereby affecting the epidemiology of Japanese encephalitis.

A recent review^34^ notes that WNV virus persists in several mammalian species, including rhesus monkeys, hamsters, mice and humans. In monkeys, hamsters and mice, virus can persist for several weeks to months in the CNS and peripheral tissues, including lymphoid tissues and kidney. Kidney targeting by WNV is related to viruria and renal pathology in human WNV-infected patients. We did not observe renal targeting by JEV, and positive RT–PCR in urine was rare. These findings indicate tropism differences between WNV and JEV.

In fact, for recurrent JEV outbreaks in temperate regions such as Hokkaido, the mechanisms of JEV hibernation are still unexplained^14^,^35^. In tropical regions, Japanese encephalitis is endemic throughout the year, as are mosquito vectors, and disease occurrence is clearly related to vector-borne transmission. In contrast, in temperate regions, Japanese encephalitis cases occur only in the warm season. Therefore, JEV re-emergence would require either reintroduction of JEV by migrating birds or a mechanism of virus overwintering in unknown hosts^3^,^35^. The re-emergence of porcine Japanese encephalitis cases in Hokkaido at the same locations indicates that JEV can overwinter locally^14^. In fact, more recent molecular analyses of JEV isolates from several genotypes present in temperate regions showed an important relationship between phylogeny and sampling location, favouring the concept of local overwintering. These studies indicate that genetic diversity of JEV isolates is driven by local virus transmission cycles rather than virus introduction from distant regions for instance by migratory birds^36^,^37^. Researchers have proposed that overwintering occurs in vertebrate hosts such as bats, in cold-blooded species or in invertebrates such as mosquitoes and ticks, the latter involving vertical virus transmission^35^,^36^,^37^. Indeed *Culex* species can overwinter locally^35^ and can transmit JEV following experimental hibernation^38^. Vertical transmission of JEV has been demonstrated experimentally in mosquitoes^39^,^40^,^41^. Nevertheless, the winter host has not been identified, despite significant effort^35^. For example, only one JEV-positive larva was found in 382,000 larvae over a period of 3.5 years in Taiwan, a fact that questions the importance of vertical transmission as an overwintering mechanism^42^. In the abovementioned areas of Hokkaido Island, in which JEV remained endemic in pigs kept in distinct areas for several years, early JEV-induced abortions were observed before mosquito season^14^. Thus, alternative transmission pathways for JEV in pigs might exist, and our results should be the basis for field studies investigating the possible persistence of JEV in pigs, as well as vector-free transmission. Future studies are now urgently required to define the impact of our findings in the light of the One Health Initiative. Despite answering questions on the occurrence of vector-free transmission and virus persistence in pigs under field situations, the cellular target of virus replication and persistence in the tonsils, the swine immune response and the impact of persistence and vector-free transmission on virus adaptation and evolution need to be investigated.

## Methods

### Animal experiment

Five animal experiments were performed under biosafety level 3 (BSL3) conditions and approved by the Cantonal Ethical Committee for animal experiments (BE 118-13). In total, 28 healthy 7-week-old Swiss Large White pigs (15 castrated males and 13 females) from our specific-pathogen-free breeding facility were used. Animals were housed in groups of ≥3 inside pens of 15 m^2^ in the containment facility of the IVI, representing a BSL3-Ag facility. Before infection, they were allowed 1 week to adapt to the new environment.

A first transmission experiment was performed in the frame of a pathogenesis study, in which three animals were infected both into the jugular vein and intradermally (i.d.) with a total dose of 10^7^ TCID_50_ of JEV (Nakayama strain, obtained from the National collection of pathogenic viruses, NCPV, Salisbury, UK) in a volume of 2 ml. The Nakayama strain is a human genotype III isolate. It was used after two passages on Vero cells (ATCC, Manassas, VA, USA). Two naive animals were kept together with the needle-infected animals to determine a possible vector-free transmission. All animals underwent necropsy at day 11 p.i.

In a second experiment, we confirmed the ability of JEV to transmit in the absence of vector by infecting two animals i.v. and i.d. as described above, and adding six naive pigs to the same pen after 24 h. The needle-infected animals underwent necropsy at day 7 p.i. and the contact pigs 9 days after being in contact with the needle-infected pigs.

In a third experiment, we determined the efficacy of oronasal infection. Nine animals were housed separately in groups of three. Each group was infected oronasally with either 10^3^, 10^5^ or 10^7^ TCID_50_ of JEV (Nakayama strain). Six animals underwent necropsy at day 10 p.i., and those infected with the highest dose were kept until day 21 p.i. In a fourth experiment, nine pigs were again housed separately in groups of three and infected oronasally with a lower dose (10^1^, 10^2^ or 10^3^ TCID_50_ of JEV Nakayama strain). For both experiments, 1 ml of infectious solution was injected carefully into different parts of the mouth without pressure using a needle-free syringe, and 1 ml was applied dropwise into the nose while holding the pig's head up.

In the fifth experiment, six animals were infected with 10^6^ TICD_50_ with a JEV genotype I strain derived from the strain JEV_CNS769_Laos_2009 strain (GenBank accession number: KC196115). It was produced as previously described using the ISA (Infectious Subgenomic Amplicons) reverse genetic method^43^. Three pigs received the virus i.d. at the base of the ear and were kept for 25 days p.i., and three pigs were infected i.v. and kept for 11 days.

The following were assessed daily in all animals: body temperature, awareness, appetite, manure excretion, breathing, gait and neurological signs. Oronasal swabs were sampled daily from each animal, and vaginal/preputial, eye and rectal swabs were taken daily from pigs of experiment three and five (Sarstedt, Nümbrecht, Germany). Blood was drawn daily using monovettes (Sarstedt). Pigs were killed by electroshock and subsequent exsanguination. Sampling was performed immediately after exsanguination. It included swabs, blood, urine, as well as organs for RT–qPCR, virus isolation and histology. The following organs were sampled: peripheral lymph nodes, tonsils, ileum, jejunum, trachea and nasal cavity. The brain was taken out *in toto*. The following CNS parts were collected: brain stem, olfactory bulb, neocortex, thalamus and basal nuclei. For histology, organ samples were fixed in 4% buffered formalin.

### Virological analyses

Organ samples were collected in 1.5-ml tubes (Sarstedt) containing 500 μl minimum essential medium (MEM; Life Technologies, Zug, Switzerland), and weighed before lysing with a BulletBlender (Next Advanced Inc., Averill Park, NY, USA). Lysed organs were centrifuged and the supernatants transferred into new tubes and frozen immediately at −80 °C. For real-time RT–qPCR, samples were thawed and spiked with enhanced green fluorescent protein RNA as an internal control, prepared as previously described^44^. RNA was extracted using a QIAmp viral RNA extraction kit (Qiagen AG, Hombrechtikon, Switzerland) according to the manufacturer's instructions. Real-time RT–PCR was performed as published^45^, using the 5′-GGTGTAAGGACTAGAGGTTAGAGG-3′ as forward primer, 5′-ATTCCCAGGTGTCAATATGCTGTT-3′ as reverse primer and FAM-cccgtggaaacaacatcatgcggc-TAMRA as probe. The RT–PCR employed the SuperScript III Platinum One-Step RT–qPCR Kit (Life Technologies) and was run on a 7900HT Thermocycler (Applied Biosystems) for 50 cycles. To ensure the quality of RNA extraction and PCR reactions, results were used only if the enhanced green fluorescent protein RT–qPCR showed a CT value <28.

Viral load was quantified relatively by using RNA from a stock of Nakayama JEV with a known titre as a standard. The stock was serially diluted 10-fold, RNA was extracted and cycle threshold (CT) values were determined to draw a standard curve, which was linear (correlation coefficient *R*=0.99) in the range of 41–12.6 CT, corresponding to 1 × 10^−1^–1.2 × 10^7^ TCID_50_ per ml of the viral stock. No amplification was obtained with CT values higher than 42. The CT value corresponding to 1 TCID_50_ was defined as 1 RNA unit. Using this standard, the CT values of our samples were transformed into relative quantities as RNA U ml^−1^. Organ samples were corrected for their weight and data calculated as relative RNA quantities in U mg^−1^.

To quantitate infectious virus, samples were serially diluted twofold in duplicates starting at a dilution of 1:2, and 100 μl of each dilution was added to confluent Vero cells (ATCC) cultured in 96-well plates with MEM supplemented with 1% fetal bovine serum (Biochrome) and 0.01 M HEPES (Life Technologies). After 4 h of incubation, the inoculum was removed and replaced with fresh medium. The cells were incubated for 72 h before fixing with 4% paraformaldehyde (Polysciences, Warrington, PA, USA) for 10 min and staining with anti-flavivirus E protein monoclonal antibody 4G2 (HB-112, ATCC) diluted in a saponin–PBS buffer (Sigma-Aldrich Chemie GmbH, Buchs, Switzerland), followed by horseradish peroxidase-conjugated goat-anti mouse antibody (Dako, Baar, Switzerland) and a final colour reaction with the 3-amino-9-ethylcarbazole (Sigma-Aldrich). Titres were calculated using the Reed and Muench formula^46^.

### Histopathology

Samples were embedded in paraffin, cut to 4-μm thickness and stained with haematoxylin and eosin. Lesions in the CNS system were semiquantitatively scored from 0 to 4 (0: no lesions; 1: minimal lesions; 2: mild lesions; 3: moderate lesions; 4: severe lesions). Scoring was performed by a blinded histopathologist and based on lymphohistiocytic perivascular cuffs, neuronal necrosis, glial nodules and parenchymal infiltration by inflammatory cells.

### Antibody responses

For plaque reduction neutralization tests (PRNT), sera were serially diluted twofold in medium in triplicate, starting at a 1:5 dilution in medium. One hundred plaque forming units per well of homologous virus were added to each well, and the serum–virus mix was gently agitated and incubated at 37 °C for 30 min. Confluent Vero cells were then incubated with the serum–virus mix for 1 h at 37 °C before washing with warm MEM (as above) and adding 200 μl 1% methylcellulose medium (Sigma-Aldrich) supplemented with 100 IU penicillin and 100 μg ml^−1^ streptomycin per well. After incubation for 48 h at 37 °C, the cells were fixed and stained as described above. As a secondary antibody, horseradish peroxidase-conjugated goat-anti pig was used at 1:500 (Bethyl, Montgomery, TX, USA). PRNT_50_ titres were read as the last serum dilution that showed a 50% plaque forming unit reduction.

### Statistical analysis

For statistical analysis, GraphPad Prism (GraphPad Software Inc., La Jolla, CA, USA) was used. To determine differences, groups were compared using a nonparametric two-tailed Mann–Whitney *U*-test setting significance to 5%.

## Additional information

**How to cite this article:** Ricklin, M. E. *et al*. Vector-free transmission and persistence of Japanese encephalitis virus in pigs. *Nat. Commun.* 7:10832 doi: 10.1038/ncomms10832 (2016).

## Figures and Tables

**Table 1:** Virological data from tonsils.

Pig No.	Infection	Virus titration* (TCID_50_ per g)	RNA quantity (RNA U per g)
1395	i.v./i.d.	3.16 × 10^3^	9.4 × 10^4^
1401	i.v./i.d.	4.39 × 10^2^	6.0 × 10^4^
1402	i.v./i.d.	6.81 × 10^4^	6.6 × 10^4^
1415	i.v./i.d.	3.16 × 10^4^	2.5 × 10^4^
1420	i.v./i.d.	>5 × 10^1^	1.0 × 10^5^
1403	Contact	6.81 × 10^3^	6.6 × 10^4^
1411	Contact	4.39 × 10^2^	1.3 × 10^4^
1416†	Contact	Negative	Negative
1417†	Contact	Negative	Negative
1418†	Contact	Negative	Negative
1421†	Contact	Negative	Negative
1422	Contact	>5 × 10^1^	1.4 × 10^4^
1423†	Contact	Negative	Negative

^*^In some samples toxic effects of the lysates reduced sensitivity (theoretically 50 TCID_50_ per g).

^†^These animals did not get infected.

**Table 2:** Virus isolation from oronasal swabs* †.

Days p.i.	Mode of infection		
	i.v./i.d.	Contact	Oronasal
1	0/5‡	0/3	0/9
2	4/11	0/3	0/9
3	3/10	0/3	6/9
4	6/7	0/3	5/9
5	8/9	0/3	5/9
6	2/3	2/2	6/6
7	0/3	1/2	2/9
8	0/3	1/2	0/9
9	—	1/2	—
10	—	1/2	—
11	—	0/2	—

^*^Theoretical sensitivity 50 TCID_50_ per ml.

^†^Results for some of the swabs were lost due to cell culture contamination and loss of material.

^‡^Number of virus-positive swabs/number of virus-negative swabs.
